# Phenotypes of a toddler with hereditary sensory and autonomic neuropathy type IV: comparing with normal: A case report

**DOI:** 10.1097/MD.0000000000036955

**Published:** 2024-01-19

**Authors:** Qinghua Xu, Yanchun Wang, Yuantao Zhou, Lu Zhang, Xiaoyi Xiang, Yucheng Xie, Jiantian Lu, Lei Li, Ying Zhu, Zhao Zhang, Tiesong Zhang, Li Li

**Affiliations:** a Kunming Key Laboratory of Children Infection and Immunity, Yunnan Key Laboratory of Children’s Major Disease Research, Yunnan Institute of Pediatrics, Kunming Children’s Hospital, Kunming, China; b Department of 2nd Infections, Kunming Children’s Hospital, Kunming, China; c Department of Pathology, Kunming Children’s Hospital, Kunming, China; d Department of Radiology, Kunming Children’s Hospital, Kunming, China; e Department of Electroencephalogram, Kunming Children’s Hospital, Kunming, China; f Department of Dermatology, Kunming Children’s Hospital, Kunming, China.

**Keywords:** congenital insensitivity to pain, genetic diagnosis, HSAN IV, magnetic resonance imaging, *NTRK1* gene, pathological analysis, whole-exome sequencing

## Abstract

**Rationale:**

Hereditary sensory and autonomic neuropathy type IV (HSAN IV) may be misdiagnosed because of low awareness among clinical professionals and overlap with other subtypes of congenital insensitivity to pain (CIP).

**Patient:**

The patient was a 1-year-and-5-months-old boy whose main symptoms were delayed psychomotor development and recurrent fever. Whole-exome sequencing (WES) revealed a compound heterozygous mutation (c. 1927C > T, c. 851-33T > A) in the *NTRK1* gene of the child. Pathological analysis showed decreased autonomic small nerve fibers, sparse hair follicles, and atrophy of the sweat glands. Sweat glands lack innervating nerve fibers. Brain magnetic resonance imaging (MRI) of the patient showed delayed myelination in the brain, slightly enlarged bilateral lateral ventricles, and patchy abnormal signals in the brain.

**Diagnosis:**

hereditary sensory and autonomic neuropathy type IV (HSAN IV).

**Intervention:**

Inform parents about the illness and take good care of the child.

**Outcomes:**

The children had less self-harming behavior and no painless fractures during follow-up at 2 years.

**Lessons:**

This report describes the pathological and imaging features and clinical manifestations of a child with HSAN IV in early life to provide a reference for the early diagnosis of the disease. Early diagnosis can help avoid self-mutilation and painless injury and reduce wound infection.

## 1. Introduction

Hereditary sensory and autonomic neuropathies (HSAN) are a group of disorders associated with sensory dysfunction (e.g., nociceptive and temperature sensation) and autonomic dysfunction (e.g., postural hypotension, gastroesophageal reflux, abnormal sweating). It was first reported by Dyck and Ohta in 1975 and was divided into 4 subtypes.^[1]^ To date, 9 subtypes of HSAN have been identified.^[2]^ Hereditary sensory and autonomic neuropathy type IV (HSAN IV), also known as congenital insensitivity to pain with anhidrosis (CIPA, MIM# 256800), is an autosomal recessive genetic disease, and pathogenic variation occurs in the *NTRK1* gene. It is characterized by extensive involvement of ectodermal structures, including the skin, skeleton, and nervous system. The main clinical manifestations are recurrent fever, analgesia, anhidrosis, mental retardation, and self-injurious behavior, which was first reported by Swanson in 1963.^[3,4]^ The pathogenic gene *NTRK1* is located on the long arm of chromosome 1 (1q21-q22), with a total length of approximately 23 kb and consists of 17 exons, encoding neurotrophic tyrosine kinase receptor type 1 (NTKR1), namely tropomyosin-related kinase A (TRKA). TRKA is a high-affinity receptor for nerve growth factor (NGF). The combination of TRKA and NGF activates downstream pathways, and plays an important regulatory role in neuronal differentiation and survival. Abnormalities in this pathway can affect neuronal growth, axon formation, and synaptic plasticity. It also renders various NGF-dependent neurons non-viable, mainly involving primary afferent neurons, autonomic sympathetic postganglionic neurons, and other neurons in the brain.^[5]^ The hereditary sensory and autonomic neuropathy type IV (HSAN IV) diagnosis mainly relies on genetic testing. However, routine next-generation sequencing (NGS) may miss the diagnosis owing to the challenges of mutation interpretation or some special variations.^[6]^ Genetic testing, in combination with other examinations, may help improve diagnosis.

CIPA is a rare disease and its worldwide prevalence is unknown. In China, 2 large cohort studies reported 41 and 36 CIPA cases, respectively.^[4,7]^ In Japan, the approximate prevalence of CIPA is 1 in 600,000-1 in 950,000.^[6]^ There is no international expert consensus for the clinical diagnosis of CIPA owing to the broad spectrum of clinical features and limited case reports. In addition to loss of pain perception, the clinical hallmarks of CIPA include varying degrees of mental retardation, self-mutilating behavior, and lack of sweating.^[6]^ When genetic testing is not available or the results are negative, histopathology and medical imaging can be used to identify clues to the disease. In this study, combined with clinical manifestations, related examinations, histopathological analysis, and genetic testing, the phenotypes of a toddler with CIPA were reported, providing clues for early diagnosis of the disease.

## 2. Materials and methods

Peripheral blood samples from the child and his parents were collected for genetic testing. Whole-exome sequencing (WES) was performed by MyGenostics Medical Laboratory (Beijing, China). The child’s skin biopsy sample (scalp) was obtained from another hospital. The scalp tissue of control child was obtained from the adjacent tissue of the scalp nevus biopsy. Immunohistochemical staining was performed by professional technicians from the hospital’s pathology department, according to the manufacturer’s instructions (Celnovte Biotechnology Co., LTD, Henan, China). Every time the patient was admitted to the hospital, routine physical examination and auxiliary examinations were performed, including serology, radiography, cranial magnetic resonance imaging (MRI), electroencephalogram (EEG), and developmental assessment. Because the brain development process of children is related to age, we selected normal brain MRI images of the same-age child as the control. This study was approved by the Ethics Committee of Kunming Children’s Hospital. Before sample collection, informed consent was obtained from the parents of the children.

## 3. Case presentations

A 1-year-and-5-months-old boy of Han nationality was admitted to the rehabilitation department of Kunming Children’s Hospital due to “delayed psychomotor development.” He was born in a non-consanguineous family with normal birth weight. Parents reported that the child had a history of asphyxia at birth. Subsequently, he was referred to the Department of Infection Diseases in the hospital because of cough and repeated fever. Subsequently, the child was hospitalized twice in the hospital rehabilitation department for rehabilitation. In addition, the patient was admitted to another hospital for skin biopsy and related examinations due to “recurrent fever.” The examination results for the patient’s several admissions are shown in Table 1.

**Table 1:** Examination results of the child.

Time of examination	Brain magnetic resonance imaging (MRI)	Electroencephalogram	Developmental assessment	Bone/joint development	The immune system	Other tests
1 year and 5 months old	Brain with slightly delayed myelination and bilateral lateral ventricles slightly enlarged	The critical point of infant electroencephalogram	Griffiths: Percentiles of the 5 dimensions of motor, personal-social, language, hand-eye coordination, and performance were all < 1%	The bilateral acetabular fossa was slightly flat and shallow.		
2 years old					CD3^+^CD8^+^: 10.67% (15–44%) CD4/CD8: 3.60 (0.7–2.8) CD19^+^: 26.49% (5–18%) IgG: 4.60 g/L (4.7–12.3) IgM: 0.45 g/L (0.47–1.75)	ALP: 103 U/L (147.7–309.3); Mg: 1.42 mmol/L (0.6–0.95); Chest CT: little pneumonia in the upper lobe of the right lung, Streptococcus pneumoniae (+) and Candida albicans (+) were detected in sputum. Whole-exome sequencing: Found no disease-associated variants.
2 years and 2 months old (examination in another hospital)			Gesell: Personal - social, adaptive, gross motor, fine motor, language, and other domains development quotient (DQ) score of 17.3 to 24.8		IgA: 0.201 g/L (0.21–1.45)	Skin biopsy (scalp): the epidermis was thinner, the dermal sweat glands were not obvious, and the collagen fibers were hyperplasia
2 years and 3 months old	There were patchy abnormal signals in the posterior horn of bilateral lateral ventricles, and the center of bilateral semiovale and bilateral lateral ventricles were slightly enlarged.	Electroencephalogram of normal children	Griffiths: Percentiles of the 5 dimensions of motor, personal-social, language, hand-eye coordination, and performance were all < 1%	The bilateral acetabular fossa was slightly flat and shallow, with moderate bone strength deficiency.	IgA: 0.14 g/L (0.21–1.45)	FT3: 4.84 pmol/L (5.2–10.2) ALP: 139 U/L (147.7–309.3) Mg: 1.07 mmol/L (0.6–0.95) Otoacoustic emission screening: TEOAE of both ears failed. Auditory brainstem response: the response threshold of the right ear was 30 dBnHL, and the response threshold of the left ear was 50 dBnHL
3 years and 1 month old		Abnormal child electroencephalogram showed increased activity of θ wave in the waking period.		There was no obvious abnormality in the bone of bilateral hip joints	IgM: 0.47 g/L (0.52–1.93) IgA: 0.24 g/L (0.3–1.88) CD19^+^: 23.57% (5–18%)	Mg: 1.12 mmol/L (0.6–0.95) Fundus photography: peripheral fundus vascularization was not complete in both eyes. Reanalysis of whole-exome sequencing data. Immunohistochemical staining.

### 3.1. Results of routine examination

When the child was 2 years and 3 months old, physical examination in the hospital’s rehabilitation department showed that 3 teeth in the right lower dentition were missing, the tip of the tongue was missing, and the muscle tension of the limbs was reduced. And his weight is 7.5 kg, which is less than −3 SD for children of the same age and sex. Previously, physical examination in the Department of Infectious Diseases revealed missing right lower canines, localized hair loss, and multiple white spots on the nails. The patient had recurrent fever due to pneumonia. During treatment, it was found that the child’s skin did not sweat, and the antipyretic drugs were not effective in lowering body temperature, but physical cooling was effective.

**Figure 1.**
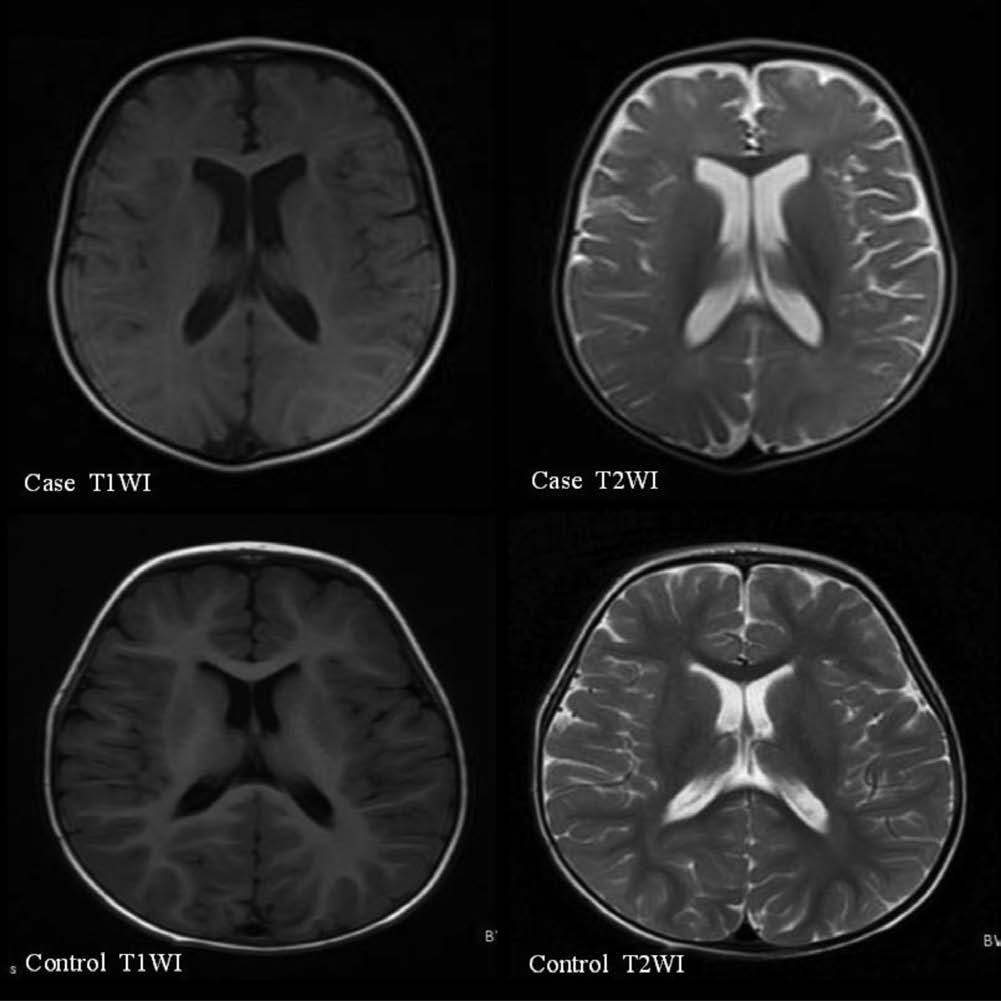
Brain MRI of the patient at 1 year and 5 months showed slightly delayed myelination in the brain and slight enlargement of bilateral lateral ventricles. MRI = magnetic resonance imaging.

**Figure 2.**
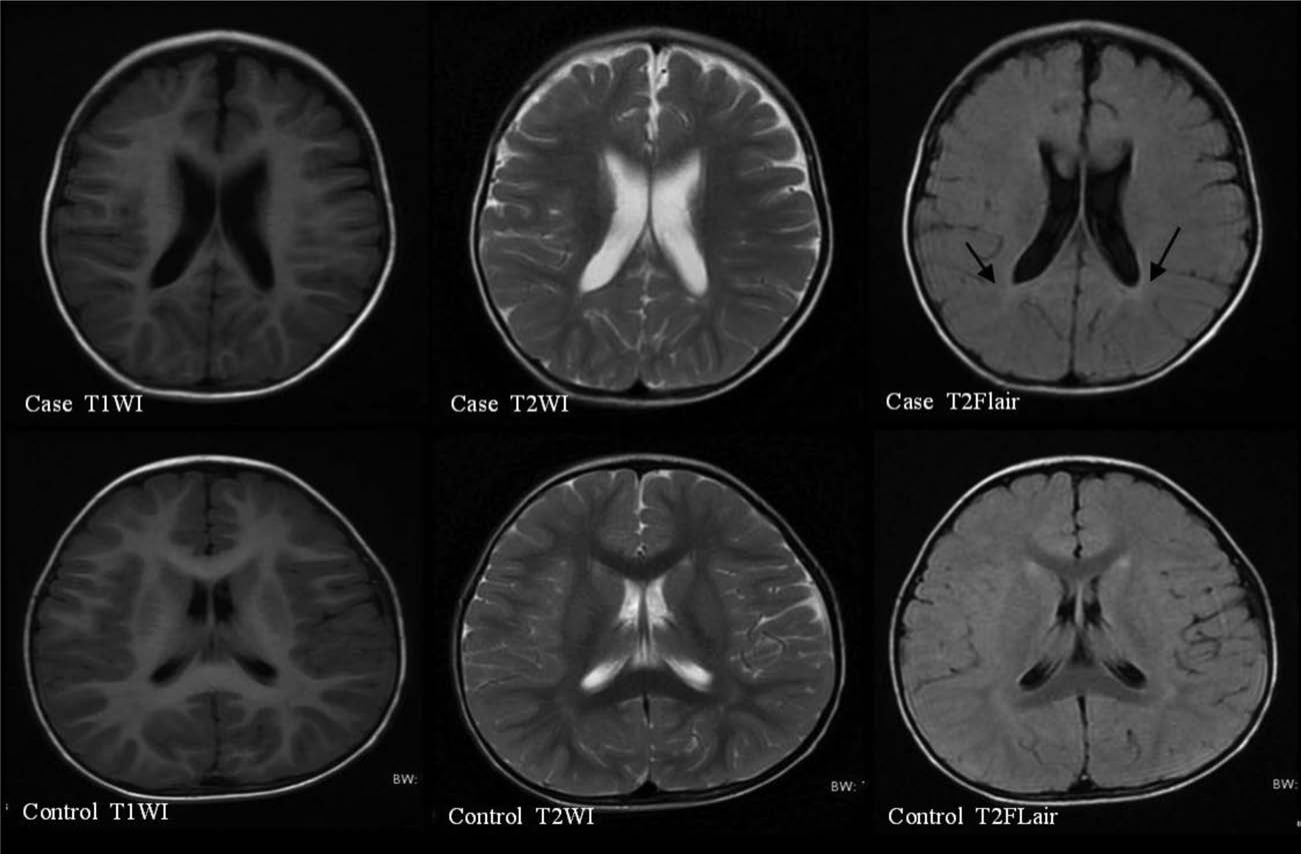
Brain MRI of the patient at 2 years and 3 months showed patchy abnormal signals in the posterior horn of bilateral lateral ventricles, with slightly enlarged bilateral lateral ventricles. MRI = magnetic resonance imaging.

**Figure 3.**
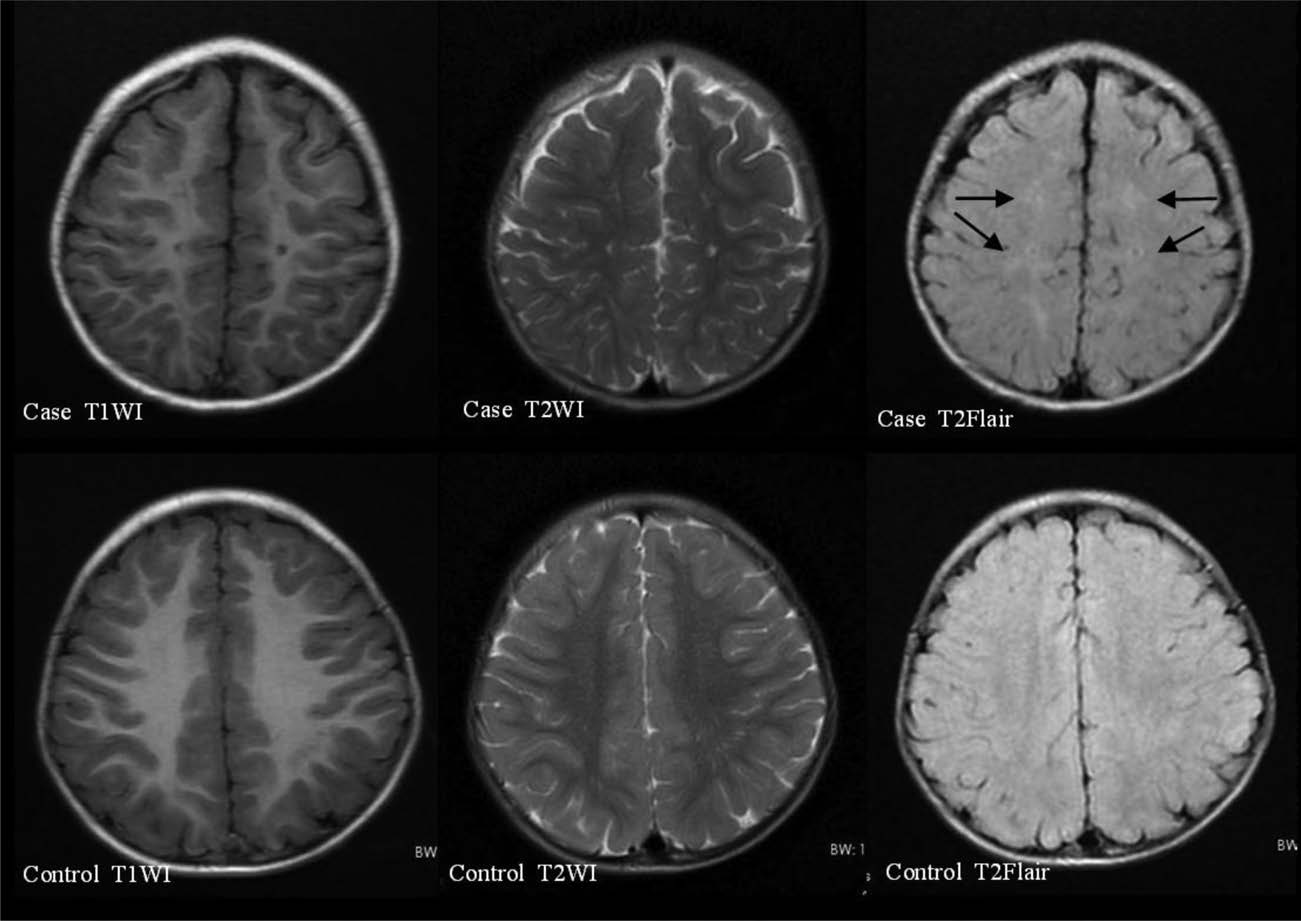
Brain MRI of the patient at 2 years and 3 months showed patchy abnormal signals in the center of bilateral semiovale. MRI = magnetic resonance imaging.

The results of immunological examinations indicated that the levels of serum immunoglobulins were low, but the ratio of B lymphocytes (CD19^+^) was increased. Radiographic examination before 3 years of age showed a slightly flat and shallow bilateral acetabular fossa. Bone mineral density examination showed moderate bone strength deficiency, accompanied by low serum alkaline phosphatase (ALP) and high serum magnesium ion (Mg2^+^) level. Brain MRI examination before 2 years of age showed slightly delayed myelination in the brain and slight enlargement of the bilateral lateral ventricles (Fig. 1), and brain MRI after 2 years of age showed patchy abnormal signals in the posterior horn of the bilateral lateral ventricles and the center of the bilateral semiovale, with slightly enlarged bilateral lateral ventricles (Figs. 2 and 3). No obvious abnormality was found in the electroencephalogram (EEG) examination of the child before age 2 years, but an abnormal EEG was found at age 3 years. Assessment of developmental milestones indicated significant delays in motor, language, adaptability, and personal-social interaction. In addition, otoacoustic emission screening showed that both ears failed, and auditory brainstem response examination revealed a response threshold of 30 dBnHL in the right ear and 50 dBnHL in the left ear. Fundus photography revealed incomplete peripheral fundus vascularization in both the eyes.

### 3.2. Results of WES

The genetic diagnosis wasn’t established until we reanalyzed the whole-exome sequencing data. The results of WES showed 2 mutations in the *NTRK1* gene of the child, c. 1927C > T and c.851-33T > A. Sanger sequencing confirmed that these 2 mutations were inherited from the mother and father, respectively, and that the child was compound heterozygous.

### 3.3. Results of histopathological analysis and immunohistochemical staining

**Figure 4.**
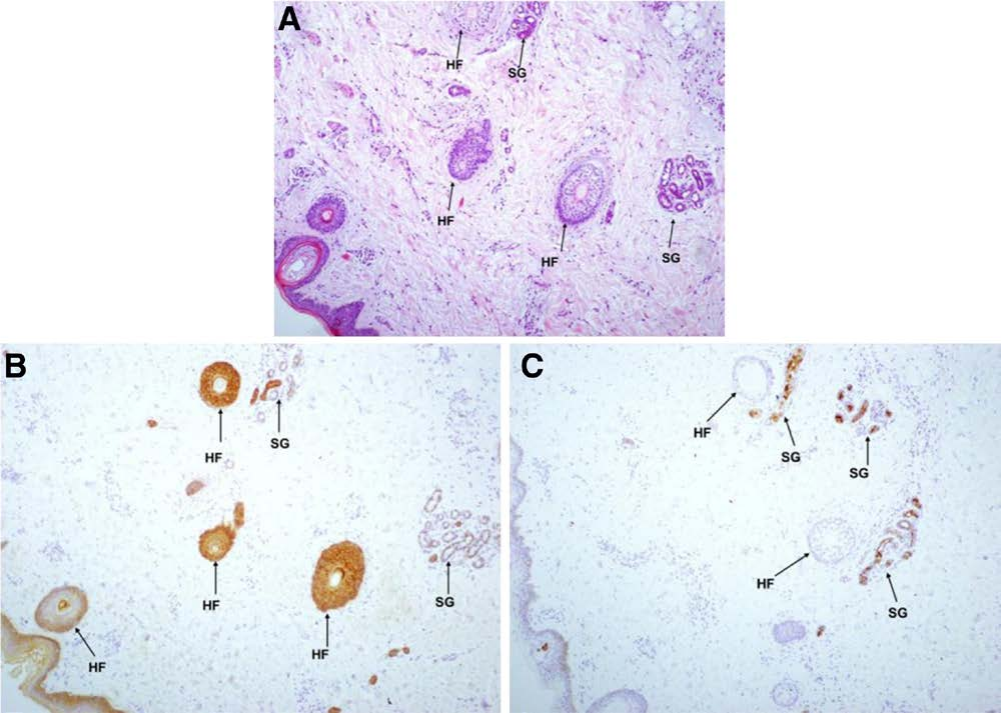
Histological analysis of the patient’s scalp revealed atrophic sweat glands and sparse hair follicles. A. The HE staining; B. The CK5/6 staining; C. The EMA staining. HF = hair follicle; SG = sweat gland.

**Figure 5.**
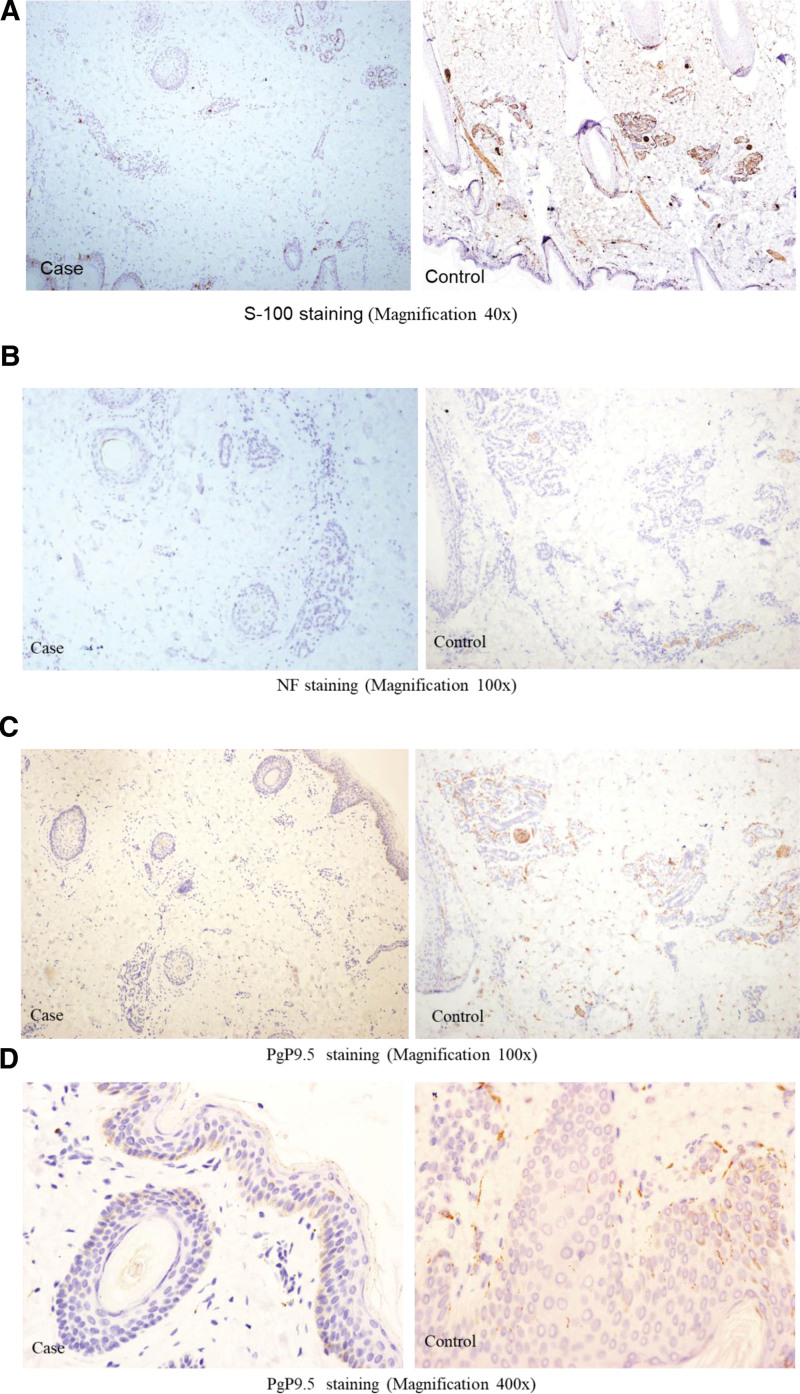
Immunohistochemical staining of the patient’s scalp. A. The S-100 staining showed that the patient’s sweat gland tissue was positive for a few myoepithelial cells, lacking nerve fiber bundles and nerve fibers innervating the sweat glands. B. The NF staining suggested a lack of nerve fiber tracts in the dermis and adjacent to the sweat gland tissue. C and D. The PgP9.5 staining showed a lack of nerve fiber bundles, scattered nerve fibers, and nerve fibers innervating sweat glands in the dermis and nerve fibers in the epidermis.

Histopathological analysis revealed atrophic sweat glands and sparse hair follicles in the dermis of the patient (Fig. 4), and the size of the sweat glands was significantly smaller than that of a normal control child (Fig. 5). S-100 staining showed that the patient’s sweat gland tissue was positive for a few myoepithelial cells, lacking nerve fiber bundles and nerve fibers innervating the sweat glands (Fig. 5A). NF staining suggested a lack of nerve fiber tracts in the dermis and tissue adjacent to the sweat gland (Fig. 5B). PgP9.5 staining showed a lack of nerve fiber bundles, scattered nerve fibers, nerve fibers innervating sweat glands in the dermis, and nerve fibers in the epidermis (Fig. 5C and D).

## 4. Discussion

CIPA is a disease involving multiple systems of the body, including the skin, bone, and nervous system. The main clinical symptoms are recurrent fever, absence of sweating, loss of pain sensation, and different degrees of mental retardation. Due to the loss of temperature sensation, pain sensation, and abnormal development of the skeletal system, the child bites off the tip of his tongue or fingers, resulting in joint dislocation, fracture, scalding, or skin damage, leading to wound infection. Early diagnosis is important to prevent injury and reduce secondary wound infections. CIPA is a rare, autosomal recessive disease. The diagnosis of this disease lacks international consensus guidelines.^[6]^ Coupled with mental retardation, some symptoms of CIPA are not easily detected, leading to difficulties in the clinical diagnosis of CIPA. For example, the parents in this report did not find that the child could not feel pain. Only after we informed the parents of the results of genetic testing did they discover that the child did not cry when he fell. We comprehensively reported the phenotype of the patient based on clinical manifestations, serological tests, imaging examinations, histopathological analysis, and genetic tests to provide a reference for the early diagnosis of the disease.

The clinical diagnosis of rare diseases is mostly based on a comprehensive analysis of clinical phenotypes and genetic test results to find clues. Previous reports have shown that most pathogenic variants of the *NTRK1* gene are single-nucleotide variants (SNVs), which can be detected by conventional WES technology. However, other genetic techniques (such as qPCR and GAP-PCR) must be used for a small number of large fragment deletions or duplications. The latter usually targets a gene or specific region when the clinical diagnosis is relatively clear.^[7,8]^ The 2 pathogenic variants we have reported (c. 1927C > T and c.851-33T > A) have been previously reported. C. 851-33T > A may be a hotspot mutation in the East Asian population.^[7]^ In vitro experiments revealed that this variant caused aberrant splicing in intron 7 resulting in a 137-base fragment insertion.^[9]^ Another variant is a missense mutation (c.1927C > T, p.R643W) that may affect TRKA protein kinase activity.^[10]^

The NGF-TRKA signaling pathway is important for the regulation of neuronal differentiation and survival. Mutation of the *NTRK1* gene causes this signaling pathway to be blocked, and various NGF-dependent neurons cannot develop and mature normally.^[5]^ The brain MRI examinations of the child were abnormal, suggesting that the child’s brain development and maturation may be abnormal, which is consistent with the results of his development assessment. Compared to cranial MRI, the sensitivity of general EEG may be lower. The EEG before the age of 3 years showed no obvious abnormalities; however, after the age of 3 years, it was found that the background θ wave activity increased during the awake period. Moreover, the specificity of EEG is poor and abnormalities may occur in intracranial infections. Intellectual development in patients with CIPA is heterogeneous. Most patients have mild-to-moderate intellectual disability, and only a few do not have obvious mental retardation.^[4]^ Some researchers evaluated the intelligence and adaptive behavior of 23 children with CIPA and found an inverse relationship between children’s age and IQ. They proposed that early intervention in these children may improve their outcomes.^[11]^

Fractures and joint dislocations are common in patients with CIPA. One of the reasons for this can be attributed to the patient’s lack of pain perception and the inability to avoid harmful stimuli. Another reason may be skeletal developmental abnormalities in the patient. Examination of the child before 3 years of age showed bilateral shallow acetabular fossa, moderate bone strength deficiency, low serum alkaline phosphatase (ALP) levels, and high serum Mg2 + levels, suggesting that the child may have abnormal bone and joint development. The tissue-nonspecific isozyme ALP in serum is mainly derived from the liver and bone, and it plays a key role in bone calcification.^[12]^ About 50% to 60% of Mg2+ in the human body is stored in bones. The concentration of Mg2 + in the blood is related to bone metabolism, and a lack of Mg2+ leads to a reduction in bone formation.^[13]^ Our patient’s serum Mg2+ level was high, but the bone density was insufficient. It is suggested that the patient may have magnesium ion transport or utilization disorders. The regulatory role of the NGF-TRKA signaling pathway in bone development requires further research. Early diagnosis and careful care can reduce the incidence of painless fractures among children.

Animal experiments have shown that the NGF-TRKA signaling pathway regulates mouse embryo blood vessel formation and ossification.^[14]^ Our examination revealed that the peripheral fundus vessels were not fully vascularized in the boy’s eyes.

Recurrent fever and anhidrosis are the primary symptoms in patients with CIPA, and skin biopsy is helpful for the diagnosis of CIPA. However, a simple morphological examination without specific immunochemical staining of the nerve tissue may also miss the diagnosis. In this case, HE, CK5/6, and EMA staining showed sparse hair follicles and hypoplasia of sweat glands. Immunohistochemical staining revealed a lack of small nerve fiber bundles and unmyelinated nerve fibers in the dermis and epidermis. Sweat glands lack nerve fiber innervation, which is consistent with earlier reports.^[15]^ This study suggests that skin nerve biopsy can be applied to confirm the diagnosis when the genetic test results are unknown, but the clinical phenotype is consistent. Human skin and subcutaneous tissues are mainly distributed with small nerve fibers with a diameter of less than 7μm, including unmyelinated C fibers and thinly-myelinated A-δ fibers, which function to conduct temperature and pain sensations and to regulate blood vessels, sweat glands, and erectus pili muscle.^[16]^ In addition to CIPA, diseases that cause small nerve fiber lesions include diabetes, poisoning, infections, autoimmune diseases, and other hereditary sensory and autonomic neuropathy types. Clinical diagnosis should be based on clinical manifestations and auxiliary examination results.^[16,17]^ In addition to these symptoms, the patient had severe malnutrition, hearing loss in both ears, recurrent diarrhea, and respiratory infections. There are few reports on deafness in patients with CIPA. In 2015, Indian researchers reported that a twin sister had sensorineural hearing loss^[18]^ suggesting that the NGF-TRKA signaling pathway may be involved in the development of auditory nerves. NGF and its receptors play bidirectional regulatory roles in the immune and nervous systems. Studies have found that NGF and its receptors may act on B cells to modulate immunity by limiting the inflammatory environment and play a role in neuroprotection, neuroregeneration, and remyelination,^[19]^ similar to our findings. We found that this patient had repeated infections, low levels of immunoglobulins, and an increased proportion of CD19^+^ lymphocytes, suggesting that the NGF-TRKA signaling pathway may be involved in the body’s humoral immune regulation. In addition, severe malnutrition the child may be associated with recurrent diarrhea.

There is no effective treatment for CIPA, and the clinical treatment is mainly symptomatic, including physical cooling for fever and anti-infective treatment when accompanied by infection. Early diagnosis and family education can effectively prevent self and external injuries. The child had less self-harming behavior and no painless fractures during follow-up at age 5 years. Rehabilitation therapy may have an effect on psychomotor developmental retardation. However, the parents did not bring the child for rehabilitation after the clinical diagnosis. The reasons may lie in the poor effectiveness of rehabilitation treatment and the financial burden.

## 5. Conclusions

We described the phenotypes of a toddler with CIPA to provide a reference for the early diagnosis of the disease. CIPA is a rare genetic disease involving the nerve, immune system, skin, bone, and other systems, and is easily misdiagnosed or missed in clinical practice. Brain MRI, skin biopsy, and immunohistochemical staining are helpful in clinical diagnosis. WES combined with clinical phenotypes helps to identify pathogenic genes. Variations in specific regions of *NTRK1* can be detected in highly suspected clinical cases. Early diagnosis and intensive monitoring can prevent trauma and self-injury and reduce wound infection.

## Author contributions

**Conceptualization:** Qinghua Xu, Tiesong Zhang.

**Data curation:** Lu Zhang.

**Funding acquisition:** Tiesong Zhang, Li Li.

**Investigation:** Qinghua Xu, Yuantao Zhou, Xiaoyi Xiang, Lei Li, Ying Zhu, Zhao Zhang.

**Methodology:** Yuantao Zhou, Yucheng Xie, Jiantian Lu.

**Project administration:** Li Li.

**Resources:** Yanchun Wang, Ying Zhu.

**Supervision:** Tiesong Zhang.

**Validation:** Yanchun Wang, Zhao Zhang.

**Visualization:** Yucheng Xie, Jiantian Lu, Lei Li.

**Writing – original draft:** Qinghua Xu, Xiaoyi Xiang.

**Writing – review & editing:** Li Li.
